# Is Ground Cover Vegetation an Effective Biological Control Enhancement Strategy against Olive Pests?

**DOI:** 10.1371/journal.pone.0117265

**Published:** 2015-02-03

**Authors:** Daniel Paredes, Luis Cayuela, Geoff M. Gurr, Mercedes Campos

**Affiliations:** 1 Departamento de Protección Ambiental, Estación Experimental de Zaidín (Consejo Superior de Investigaciones Científicas), Granada, Andalucía, Spain; 2 Departamento de Biología y Geología, Universidad Rey Juan Carlos I, Móstoles, Madrid, Spain; 3 Graham Centre for Agricultural Innovation (New South Wales Department of Primary Industries and Charles Sturt University), Orange, New South Wales, Australia; University of California, Berkeley, UNITED STATES

## Abstract

Ground cover vegetation is often added or allowed to generate to promote conservation biological control, especially in perennial crops. Nevertheless, there is inconsistent evidence of its effectiveness, with studies reporting positive, nil or negative effects on pest control. This might arise from differences between studies at the local scale (e.g. orchard management and land use history), the landscape context (e.g. presence of patches of natural or semi-natural vegetation near the focal orchard), or regional factors, particularly climate in the year of the study. Here we present the findings from a long-term regional monitoring program conducted on four pest species (*Bactrocera oleae*, *Prays oleae*, *Euphyllura olivina*, *Saissetia oleae*) in 2,528 olive groves in Andalusia (Spain) from 2006 to 2012. Generalized linear mixed effect models were used to analyze the effect of ground cover on different response variables related to pest abundance, while accounting for variability at the local, landscape and regional scales. There were small and inconsistent effects of ground cover on the abundance of pests whilst local, landscape and regional variability explained a large proportion of the variability in pest response variables. This highlights the importance of local and landscape-related variables in biological control and the potential effects that might emerge from their interaction with practices, such as groundcover vegetation, implemented to promote natural enemy activity. The study points to perennial vegetation close to the focal crop as a promising alternative strategy for conservation biological control that should receive more attention.

## Introduction

Habitat management is a conservation biological pest control strategy focused on manipulating the environment to enhance natural enemy populations [1], [2]. A common conservation biological control practice in perennial crops is the establishment and management of a ground cover [3], which often consists of an inter-tree herbaceous vegetation strip, although it can extend as a continuous covering across the crop [4]. Ground cover can be formed by single species or a polyculture, with species typically belonging to Poaceae and Fabaceae [5], [6] and other herbs [7], [8] and is often comprised of self-regenerating vegetation [9], [10]. Ground cover can improve the diversity of vegetation, especially if replacing bare ground, and creates new habitat structure [1]. As a result, the abundance [9] and diversity of natural enemies increase [8], [11], which might promote higher rates of predation and parasitism of insect pests by natural enemies [12]. The identity of ground cover species can however benefit some pests, so ‘selective plants’ have been recommended such that only natural enemies derive benefit [13].

Despite any increase in the abundance of predatory and parasitoid insects led by the use of ground covers, it is still uncertain whether this will translate into reduced pest densities. Much of the existing literature focuses on the effects of ground covers on natural enemies, implicitly assuming that an increase in their abundance or diversity will necessarily lead to a decrease in pest abundance. However, some studies have demonstrated that this does not consistently occur, as intraguild predation and other biotic processes can diminish the impact of natural enemy species assemblages on herbivore populations [14–16]. Further, those studies that explicitly investigate the effects of ground cover on herbivore abundance provide inconsistent evidence ([17] and references herein). For example, according to different studies ground cover in peach orchards could either decrease [18], [19] or increase the abundance of pests [5], [20]. Their use in other orchards (apple, pear, citrus, olive) has yielded likewise either positive or nil results in terms of biological control (see S1 Appendix for a comprehensive review of the literature).

Inconsistent findings might arise from differences in local conditions (e.g. crop management, land use history), the landscape context (e.g. surrounding natural or semi-natural vegetation), and variables operating at a regional scale (e.g. climatic conditions of the region at the time when the study was conducted) [1], [21]-[23]. The influence of factors operating at these different scales is often not apparent in studies conducted at one single locality and that have no representation of heterogeneity at other scales or temporal replication. In this study we present evidence from a long-term (2006–2012) regional assessment conducted in 2,528 olive orchards in the Andalusia region, Spain, in order to assess the effect of ground cover on the abundance of pest herbivores. Through this research, we aim to provide a robust conclusion on the utility of ground cover in olive orchards as well as to tease-apart the relative importance of other unmeasured effects over a range of scales (local, landscape, regional). Our study is unique in terms of the large spatial (2,528 orchards) and temporal (seven years) replication of samples using the same methodology, which allows detection and quantification of the effect of ground cover on different pest species, while accounting for non-specific random variance at the local, landscape and regional scale.

## Materials and Methods

### Data collection

**Figure 1:**
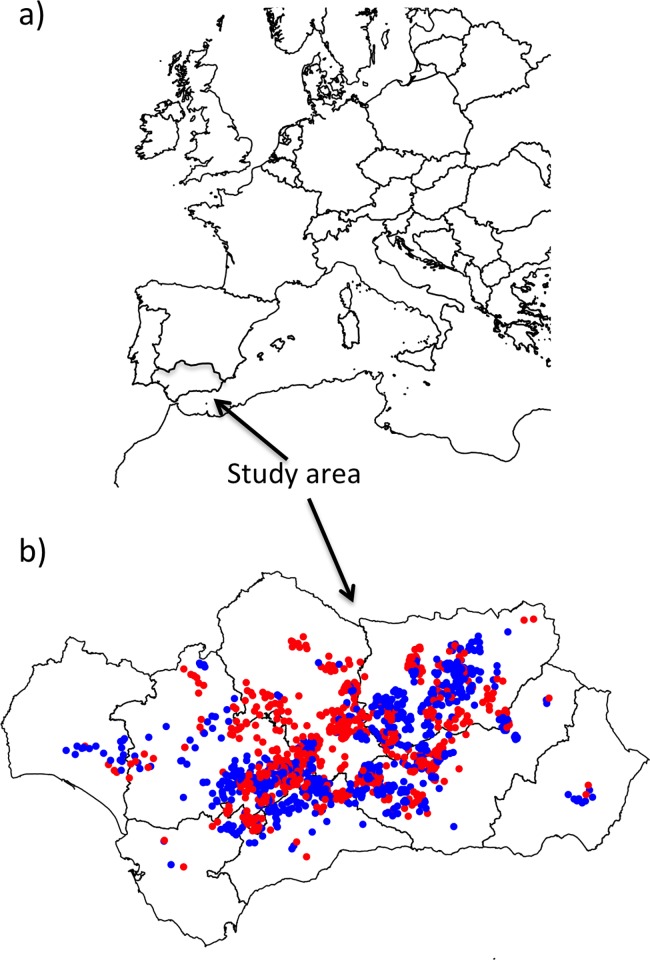
Map of the Andalusian region, Spain, and the monitoring stations used in this study. Red points represent ground cover MS while blue points represent bare soil MS before pairing.

The Warning and Information Plant Protection Network (WIPPN) of Andalusia (Spain) monitors the populations of potentially dangerous arthropods and provides guidelines to farmers on how to treat pests. It has several monitoring stations (MS) spread throughout the region, each covering an area of ca. five hectares. These are part of private olive groves that have an agreement with the administration to monitor pests. All these farms are handled under integrated protection management criteria (IPM) so insecticide treatments are made whenever a pest population exceeds relevant threshold levels. Since 2006, technicians of the WIPPN have collected data on olive pest numbers, together with information about the orchards, including presence of ground cover vegetation following a prescribed protocol [24] (Fig. 1). Ground cover vegetation in these sites is comprised of naturally generating plants, chiefly grasses interspersed with broadleaved plants. No detailed data exist for botanical composition of the groundcovers so, for the purposes of this analysis, groundcovers were not categorised. In this work we use data collected by the WIPPN from 2006 to 2012 to investigate the effects of ground cover on four of the most common pests found in this crop: *Prays oleae* (Bern.) (Lepidoptera: Yponomeutidae) (the olive moth), *Bactrocera oleae* (Rossi) (Diptera Tephritidae) (the olive fly), *Euphyllura olivina* Costa (Hemiptera: Psyllidae) (the olive psyllid), and *Saissetia oleae* (Olivier) (Hemiptera: Coccidae (the olive scale).

***Prays oleae***. The olive moth is one of the principal insect pests in olive groves. The damage caused by this insect can reduce olive production at a level of 50–60%, which equates to 8–11 kg per tree in modern cultivars [25]. It has three generations: phylophagous (leaf generation), anthophagous (flower generation), and carpophagous (fruit generation) [26]. The two latter are the most damaging. The leaf generation overwinters inside the leaves and is not very abundant. The flower generation is the most abundant of the three, and attacks the floral buttons, drastically reducing the potential fruit set. The larvae of the fruit generation, which develop inside the olive stone, cause its premature fall, decreasing crop productivity.

To measure abundance of adults, two funnel traps, baited with the sex pheromone Z-7 tetradecenal, were located in every MS and were checked every week from the beginning of March to the end of November (Table 1). A major issue is that these traps catch simultaneously adults from the three generations. The leaf and flower generations partly overlap through time, but can be set apart from the fruit generation across time. In addition, a total of 400 inflorescences were collected from mid March to the end of May at each MS, and percentage of inflorescences with larvae was used as a proxy to population size of the flower generation. Similarly, a total of 400 olive fruits were taken from the beginning of August to the end of September at each MS, and the percentage of fruits with larvae of the fruit generation was calculated (Table 1). Percentages were transformed to number of successes (infested) and failures (not infested) for the corresponding response variables, over a total of 400 inflorescences and fruits respectively.

**Table 1:** Response variables used for each of the four species analysed in this study, family error used in the generalized linear mixed models, and number of observations (i.e. number of monitoring stations) available for each response variable for all the years.

Species	Response variable	Type of response variable	GLM error distribution	Number of MSs used
*P. oleae*				
	Funnel traps adults generation 1&2	Count	Negative binomial	2418
	Larvae / inflorescence	Proportion	Binomial	1984
	Funnel traps adults generation 3	Count	Negative binomial	1114
	Larvae / fruit	Proportion	Binomial	2160
*B. oleae*				
	Funnel traps adults	Count	Negative binomial	2118
	Sticky traps adults	Count	Negative binomial	2528
	Damaged fruits	Proportion	Gaussian	754
*E. olivina*	Nymphs / inflorescence	Proportion	Binomial	276
*S. oleae*	Living forms / shoot	Count	Negative binomial	354

***Bactrocera oleae***. The olive fruit fly oviposits in the olive fruit mesocarp, where larvae develop through three instar stages [26] depending on environmental conditions, from July to late autumn, the most harmful being that occurring in autumn. Tunnelling and falling of fruits caused by the larvae of the olive fruit can be very high, causing in some years the almost complete loss of olive production, where no pest management measures are undertaken. Populations of this insect were estimated weekly at each MS from the beginning of June to the end of December, by means of three Mc-Phail traps and three sticky traps baited with the pheromones diammonium phosphate and dioxaspiro [5.5] undecane, respectively. The damage caused by the olive fly was also estimated by counting the proportion of fruits damaged by this insect during the same period as for sticky and Mc-Phail traps (Table 1).

***Euphyllura olivine***. The olive psyllid is a major pest in North Africa [27]. In European Mediterranean countries it is usually considered a secondary pest but could become a more serious threat to olive crops as a result of climate change which would cause a shift of the species range northwards in Europe [28]. The nymphs of this insect attack the young shoots and inflorescences causing damages to fruits (30%) and inflorescences (70%) [27]. It has three or four generations per year, the first being the most damaging. From mid March to the end of May, the number of inflorescences, out of 400 per MS, which had nymphs of olive psyllid was used to estimate the damage caused by this insect (Table 1).

***Saissetia oleae***. The olive scale is widely distributed and attacks several fruit trees. This pest infests leaves and twigs, sucks the tree sap and produces large amounts of honeydew, which serves as a substrate for sooty mould fungi; the latter interferes with photosynthetic and respiratory processes of the plant and affects the quality of the fruit. This results in yield decreases and rejection of fruit for export [29]. To quantify the presence of this pest, the total number of individuals caught in 200 shoots was counted weekly at each MS during June (Table 1).

### Data processing and analyses

For each pest species we selected response variables that represent—directly or indirectly—population size-related measures and for which there were enough observations (> 250) in the database to conduct reliable statistical analyses (Table 1).

We used the maximum value attained throughout the year for response variables that were measured weekly. We chose peak of abundance instead of cumulative abundance, as there was a high variability in the number of observations per orchard. The dates of first and last assessment and duration of data collection (i.e. number of samples per growing season) differed between sites, years and response variable. This precluded analysis of total or cumulative pest values because site and year data were not comparable. Accordingly, we analysed peak values for each variable to check whether using peak rather than cumulative numbers distorted measures of pest metrics. We correlated peak abundance with cumulative abundance for data sets where there was a consistent number of observations. Correlations between peak of abundance and estimated cumulative abundance for all response variables were always high (minimum r = 0.773) (S2 Appendix).

To account for variability at the landscape scale, we compared paired samples of MSs with ground cover and bare soil. Pairing was performed by selecting for each monitoring station with ground cover vegetation the nearest monitoring station with bare soil (up to a maximum distance of 10 km). We repeated this procedure for all MSs until there were no more possible pairs and disregarded data from remaining unpaired sites. Pairing was also constrained within cultivars in order to maintain as much homogeneity as possible. We repeated the pairing every year because not all MSs had data available every year. Consequently, the number of observations used for analysis was not constant for each response variable (Table 1). Because of that, we avoided to use a time series framework to include the variable year. Doing this would force us to withdraw data from many monitoring stations, weakening the statistical power of our models. Therefore, we considered year as a geographic regional influence because: (1) all data taken within a given year share similar macroclimatic conditions; (2) if macroclimatic conditions have an influence on pest abundance, we are effectively modelling such effects on the response variable (i.e. pest abundance) by incorporating year as a random effect.

We used generalized linear mixed models (GLMMs) to analyse the effect of ground cover on the different response variables by accounting for random effects at different spatial scales: plot, pair, and year. Depending on the nature of the response variable and post-hoc evaluation of model residuals we used different error distributions. A Gaussian error distribution was used for raw proportions (e.g. 75% of fruits damaged by *B*. *oleae*). A negative binomial error distribution was selected for count data, whereas a binomial error distribution was chosen for proportions where we had information on the number of successes (infestation) and failures (no infestation) of a particular event (e.g. 300 fruits with and 100 fruits without larvae of *P*. *oleae*) (Table 1). Random factors were used to model potential structures of autocorrelation in the dataset and were attributed to unmeasured features that can be found at different scales, specifically: (1) 'plot' represents local scale variability for MS that have been sampled at different years, and might thus have a similar response throughout time due to crop management related issues such as intensity of pesticide application, land use history, topography or micro-climatic conditions or even different types of ground cover that might have an important effect on pest abundance [13], [30]-[32]; (2) 'pair' refers to the geographical pairing of MS. Paired MS might share a common response to pests, regardless they have ground cover or bare soil, due to landscape-scale factors such as crop diversity, landscape diversity, landscape complexity or presence of patches of natural or semi-natural vegetation and their related measures such as mean patch size, edge density, and so on [21], [33], [34]; and (3) 'year' represents particular macro-climatic conditions such as temperature and humidity. This climatic features might equally affect all plots at the regional level since it is well known that some years are climatically more suitable for pest outbreaks than others [22], [35].

We built all possible combinations of random and fixed factors. 'Pair' was nested within 'year', but plot was not nested within 'pair', because the assignment of each 'plot' to a 'pair' might change from one year to the next. Overall we fitted 15 models that were compared using the Akaike Information Criterion (AIC). Models with a difference in AIC > 2 indicate that the worse model has virtually no support and could be omitted. Following Nakagawa and Schielzeth [36], we calculated the R^2^ to have an account of the variability supported by models with Gaussian or binomial error distribution. Though this approach cannot be applied to models with a negative binomial error distribution (S. Nagakawa, personal communication), it allows two components of R^2^ to be calculated: (1) a marginal R^2^ $\left(R_{m}^{2}\right)$ that only takes into account the variability explained by fixed effects; and (2) a conditional R^2^ $\left(R_{c}^{2}\right)$ that accounts for the variability supported by both the fixed and random effects. When ground cover was included in at least one of the best models, we quantified its effect in relation to the different sources of random variability (local, landscape and regional) for each response variable. To achieve this, we calculated the difference between ground cover and bare soil from the fixed effects estimated model parameters, and added a random error from the corresponding variance term estimated by the model at each scale of variability. We repeated this procedure 1000 times and plot the 95% confidence intervals of such predictions.

All the analyses were performed in R [37], and its packages’lme4' [38] and “glmmADMB” [39].

## Results

Model selection indicated that there were either one or two best models for each pest response variable. In all cases, except for *E*. *olivina* and *S*. *oleae*, at least one of the best models included the fixed and all the random effects (Table 2). Alternative best models for some response variables (*P*. *oleae* funnel traps in generation 3; *B*. *oleae* funnel traps and fruit damage; *S*. *oleae*, just with regional as random factor selected) excluded the fixed effect of ground cover. For *E*. *olivina*, the best model excluded the regional-scale random effect (Table 2). *S*. *oleae* displayed four best models, all including regional factor as random effect but also the combination of this with local and landscape factors separately. For all best models for which it was possible to calculate the R^2^, we found a much higher conditional $\left(R_{c}^{2}\right)$ than marginal R^2^ $\left(R_{m}^{2}\right)$ (Table 2).

**Table 2:** Comparison of alternative models (using AIC_c_) for the response variables tested in the study.

Model		Species and response variable									
		*P. oleae*				*B. oleae*			*E. olivina*	*S. oleae*	
		Funnel traps generation 1&2	Funnel traps generation 3	Larvae/inflorescence	Larvae/fruit	Funnel traps	Sticky traps	Damaged fruits	Presence/inflorescence	Presence/shoot	
Fixed effect	Random effects										
Ground cover	No	41931.20	14938.70	59937.78	212352.6	22579.60	32829.80	5151.03	8841.59	3168.52	
	Local	41090.40	14724.66	18800.90	86147.74	22082.00	32562.00	5131.30	2491.27	3170.52	
	Landscape	40940.20	14607.88	16803.58	69063.21	21970.20	32542.40	5064.25	2015.06	3168.68	
	Regional	41510.80	14637.70	45024.71	187061.4	22313.20	32204.60	5049.47	7223.38	**3165.70**	
	Local+Landscape	40668.60	14594.02	7935.64	23439.52	21795.40	32396.40	5045.40	**1294.32**	3170.68	
	Local+Regional	40656.00	14493.26	16280.28	77530.91	21628.60	32203.20	5023.11	1673.95	**3167.70**	
	Landscape+Regional	40732.60	14430.82	16661.74	68987.29	21753.00	32371.00	4995.03	2009.70	**3167.40**	
	Local+Landscape+Regional	**40398.20**	**14411.26**	**7803.70**	**23397.89**	**21507.80**	**32128.60**	**4978.63**	1297.49	3169.40	
No ground cover	Local	41094.20	14723.68	18799.81	86150.07	22080.20	32563.60	5130.32	2502.77	3171.88	
	Landscape	40943.80	14607.50	16841.12	69461.36	21968.40	32545.20	5063.33	2026.55	3171.80	
	Regional	41508.80	14635.70	45059.58	187405.7	22316.80	32580.20	5048.25	7233.54	**3167.28**	
	Local+Landscape	40712.12	14592.90	7940.64	23466.91	21794.40	32401.00	5044.49	1300.44	3172.80	
	Local+Regional	40658.00	14491.78	16298.67	77539.27	21627.00	32205.40	5021.93	1683.19	3169.28	
	Landscape+Regional	40735.20	14430.04	16699.28	69385.44	21751.00	32374.40	4994.11	2021.19	3169.20	
	Local+Landscape+Regional	40401.00	**14409.88**	7810.15	23424.78	**21506.60**	32132.60	**4977.29**	1303.72	3172.80	
	$\left(R_{m}^{2}\right)$	N.A.	N.A.	0.00165	0.00562	N.A.	N.A.	0.00046	0.01245	N.A.	
	$\left(R_{c}^{2}\right)$	N.A.	N.A.	0.41615	0.56952	N.A.	N.A.	0.62922	0.73679	N.A.	

The best model (lowest AIC_c_) is indicated in boldface type. The marginal (m) and conditional (c) R^2^, when its calculation was possible, refer to the best model.

**Figure 2:**
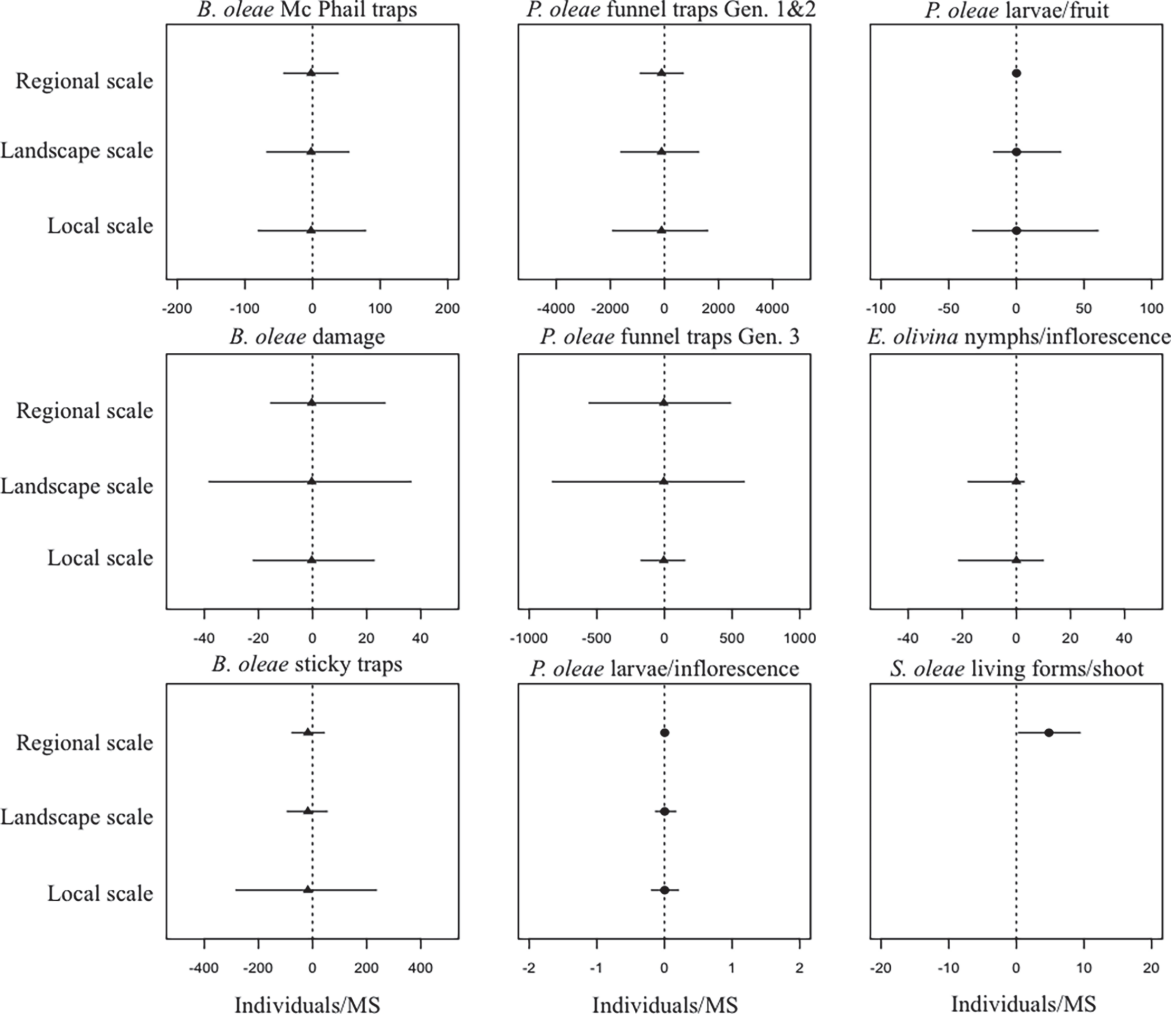
Best model estimations for the differences between ground cover and bare soil for all the response variables. Positive values represent an increase in pest abundance in the presence of ground cover (filled circle) whereas negative values represent a decrease in pest abundance in the presence of ground cover (filled triangle). Bars represent a 95% of the confidence interval of the random effects predictions (± σ^2^) at a local, landscape, and regional scale. If a random factor is not included in the selected best model (Table 2), no 95% confidence interval bars were drawn.

Model estimations showed that, even if ground cover was included in the best model, it had a minor effect in absolute terms compared to random variability. This was particularly the case for local (i.e. plot) and landscape (i.e. pair) scales, although regional variability was also important for most of the response variables. However, for *S*. *oleae* random variability associated with factors at the regional scale reported an effect comparable to ground cover (Fig. 2).

## Discussion

The results of this study indicate that ground cover, as a single entity and compared to bare ground, is not an effective measure to reduce pest abundance in olive groves. This is evident in the absence of this term in some of the best fit models, the low amount of variability explained by this factor in those models where it was present, and its small absolute effect—as quantified by estimated model parameters—in relation to the random factors. In contrast, random effects, explained a greater proportion of the observed variability in all response variables. Such factors represent the effect of variables operating at different spatial and temporal scales, whose variability has not been specifically measured, and which might have an influence in the response variable. Although the processes operating behind random factors are unknown, here we provide an account of some of the potential variables that might be explaining the high variability observed in pest abundance or related response variables (e.g. pest damage to crop) at different spatial scales.

At the local scale, crop management related issues such as intensity of pesticide application, land use history, topography or micro-climatic features, might have an important effect on pest abundance. The botanical composition of ground cover [40], its density [41], the width of the strip [42], or the height of grasses might also have an influence on pest abundance [43]. The lack of data on any variability in groundcover types in the available data set, however, precludes testing for any optimal forms of vegetation or management. The abundance of *B*. *oleae* has been demonstrated to be influenced by site elevation [30]. Another factor that could influence pest populations is the history of pesticide use [31] and the nature of the active constituent in insecticides [31]. Intensive use of pesticides, particularly broad spectrum types could depress the local community of natural enemies thereby favouring herbivores independently of direct effects on pest populations. Further, any lack of nearby non-crop vegetation would increase the detrimental effects of pesticide, as natural enemies will have no refuge from which to recolonize sprayed crops [17], [33], [44].

Landscape-scale factors relate largely to the composition and connectivity of vegetation including other crops, natural or semi-natural vegetation, at ranges up to 10 km from the crop that are known to affect the presence of natural enemies in crops [21], [33], [34]. In Andalusia, as well as in other Mediterranean regions, olive groves can adopt a variety of forms, from highly intensified crops where landscape heterogeneity is very low, to less intensively managed crops in areas with high landscape heterogeneity, particularly in mountainous areas [45]. In olive groves, areas of herbaceous vegetation and areas of woody vegetation near olive crops, and smaller patches of woody vegetation within olive groves, decreased the abundance of *P*. *oleae* and *E*. *olivina* [47]. Landscape-related features such as mean patch size, edge density, and landscape diversity, might likewise help reduce *B*. *oleae* abundance [48], [49]. This does not necessarily mean that the more the natural vegetation the more effective the biological control. Indeed, some studies seem to point out to complex interactions between different landscape and local features [50], [51], sometimes reinforcing their mutual role in biological control, and some other times counteracting their effects [52]. For example, when both habitats were present in an olive crop, the abundance of some groups of natural enemies (spider and parasitoids) was positively influenced by adjacent vegetation, whereas this effect was lower or even reversed in bare soil plots [50]. Habitats provided by surrounding natural vegetation can also produce the opposite effect by removing, rather than providing, natural enemies to the crop [53]. Overall, these landscape scale variables are well known to have an influence on pest abundance in crops [21], [33], [46] so it was not our aim to specifically test for such effects. Rather, this study assessed the relative impact of groundcover vegetation in relation to other variables that would be operating at the scale of landscape as well as more local and regional scales.

At a regional scale, inter-annual climatic variability might have a major influence on pest abundance. Changes in temperature and humidity could alter the phenology of pests and natural enemies and, therefore, influence insect population growth rate [22], [35]. Humidity can affect herbivores such as *S*. *oleae*, as larger populations of this insect have been found near rivers or creeks [54]. This would explain why the amount of variability explained by this factor was higher for *S*. *oleae* than for other species. In a recent study in olive groves [47], we detected a high variability in the response of *E*. *olivina* and *P*. *oleae* to ground cover and different surrounding natural vegetation features between two consecutive years, and attributed these changes to climatic variability between years.

From a farmer perspective, local conditions are too varied to be accounted for when outlining pest control strategies. Similarly, nothing can be done to account for inter-annual climatic variability, even thought the response of pests to any treatment, including ground cover, might be dependent on climatic conditions [35]. Landscape-scale factors, on the other hand, can be taken into account by farmers and are thus subjected to be included in the design of strategies against pests. Farmers could potentially manage vegetation at a whole-farm scale to provide features that promote biological control such as refuge habitat for natural enemies. Farmers could also site production areas on parts of their property to best benefit from any vegetation beyond their ownership boundary that might provide similar benefits. Thus, future research should pay more attention to the landscape context, particularly to perennial non-crop vegetation surrounding or nearby the crop, and to the interactions between these structures and ground cover. Although our study has demonstrated that ground cover by itself is not particularly efficient in terms of biological control, investigating different kinds of vegetation and management practices could identify particular options that do strongly promote biological control. Crucially, however, this analysis of a large data set indicated clearly that groundcovers of the type that is common in Spanish olive groves does not strongly promote biological control. Finally, we acknowledge that other ecosystem services, such as soil fertility and prevention of soil erosion [55], [56], or pollination enhancement [57], can be important so need to be considered in future design of functional groundcovers.

## Supporting Information

S1 Appendix Review on works dealing with ground cover. Based on and expanded from Simon et al. (2010). The effect of ground cover on pest control is considered to be positive, null or negative when either the density of the pest arthropod of the fruit tree and fruit damage is lower, equal or higher, respectively, compared with control.
(DOCX)

S2 Appendix Statistical descriptors of the number of observations and correlations between peak and cumulative abundance. (DOCX)
