# Supplementary material for: Is Ground Cover Vegetation an Effective Biological Control Enhancement Strategy against Olive Pests?
Source: PLoS One. 2015 Feb 3;10(2):e0117265. doi: 10.1371/journal.pone.0117265 (PMC4315409; doi:10.1371/journal.pone.0117265)
Supplement: S1 Appendix — Based on and expanded from Simon et al. (2010). The effect of ground cover on pest control is considered to be positive, null or negative when either the density of the pest arthropod of the fruit tree and fruit damage is lower, equal or higher, respectively, compared with control. (DOCX) [file pone.0117265.s001.docx]

Appendix S1

| Crop type | Pest | Ground cover treatments | Effect on pest | Source/Region |
| --- | --- | --- | --- | --- |
| Citrus | *Frankinella occidentalis* and *Thrips tabaci* | Wild, planted (*Festuca arundinacea*) and bare soil | Positve planted, Negative wild and bare soil | Aguilar-Fenollosa and Jacas, 2013/Spain |
| Citrus | *Tetranychus urticae* | Wild, planted (*Festuca arundinacea*) and bare soil | Positive planted | Aguilar-Fenollosa et al., 2011/Spain |
| Citrus | Hemiptera | Wild | Null | Stevens et al., 2007/Australia |
| Olive | *Prays oleae* and *Euphyllura olivina* | Wild and bare soil | Null | Paredes et al., 2013/Spain |
| Olive | *Prays oleae* and *Bactrocera oleae* | Cereal | Variable | Rodriguez et al., 2009/Spain |
| Peach | *Lyonetia clerkella* | *Medicago sativa* and wild natural cover | Positive *Medicago* | Dong et al., 2005/China |
| Peach | *Aromia bungii* and *Halyomorpha halys* | *Trifolium repens* and bare soil | Negative | Wan et al., 2011/China |
| Peach | *Tetranichus urticae* | Wild, bare soil, *Vicia angustifolia* | Negative with *Vicia angustifolia* | Meagher and Meyer, 1990a/USA |
| Peach | Leafhopper vectors of X-disease | *Trifolium pretense*, mixed of rosaceous species, *Dactilys glometrata* and bare soil | Negative | McClure et al., 1982/USA |
| Peach | Hemipterans | Bare soil, strip sod and solid sod | Negative with sod | Meagher and Meyer, 1990b/USA |
| Apple | Apple pests | Wild | Positive | Altieri and Smith, 1985/USA |
| Apple | Spider mites | Wild | Null | Nyrop et al., 1994/USA |
| Apple | *Dysaphis plantaginea* and *Aphis pomi* | Weed strips and bare soil | Positive | Wiss, 1995/Switzerland |
| Apple | Leafroller | *Fagopyrum sculentum* | Positive | Stephens et al., 1998/New Zealand |
| Apple | Apple pests | Wild | Null | Jenser et al., 1999/USA |
| Apple | Leafroller | *Lobularia maritima*, *Phacelia tanacetifolia* and *Fagopyrum esculentum* | *Fagopyrum* Positive, *Phacelia* Positive, *Lobularia* Negative | Irvin et al., 2006/USA |
| Apple | Aphis spp. | Planted, wild, mowed grass and bare soil | Null | Marko et al., 2013/Hungary |
| Apple | *Helicoverpa armigera* | Severals seed mixes | Null | Bone et al., 2009/Australia |
| Apple | Aphis spp. | *Phacelia tanacetifolia*, *Fagopyrum esculetum* and a mix of Poaceas species | Null | Frechette et al., 2008/Canada |
| Vineyards | *Erythroneura alegantula* and *Frankliniella occidentalis* | *Fagopyrum esculentum* | Positive | Nicholls et al., 2000/USA |
| Vineyards | *Erythroneura* spp. | Wild and bare soil | Null | Costello and Danne, 2003/USA |
| Vineyards | *Erythroneura* spp. | *Fagopyrum esculentum*, *Trifolium repens* and *Dactylis glomerata* | Null | English-Loeb et al., 2003/USA |
| Vineyards | Tortricidae | *Fagopyrum esculentum* | Null | Berndt et al., 2006/New Zealand |
| Fraser Fir Christmas tree | *Oligonychus ununguis* | *Trifolium repens*, *Trifolium pretense*, *Lotus corniculatus*, *Festuca* spp. | Null | Willians et al., 2011/USA |
| Pear | *Panonychus ulmi* (in apple) or *Cacopsylla pyricola* (in pear) | Flower plant mixtures | Null | Fitzgelard and Solomon, 2004/UK |
| Pear | *Cacopsylla pyri* | Planted, wild and bare soil | Positive | Rieux et al., 1999/France |
| Pear | *Psylla chinensis*, *Aphis citricola* and *Pseudococcus comstocki* | Aromatic, wild and bare soil | Positive | Beizhou et al., 2011 |

**REFERENCES Appendix S1**

1. Aguilar-Fenollosa E, Jacas JA (2013) Effect of ground cover management on Thysanoptera (thrips) in clementine mandarin orchards. J Pest Sci 86: 469-481.
2. Aguilar-Fenollosa E, Pascual-Ruiz S, Hurtado MA, Jacas JA (2011) Efficacy and economics of ground cover management as a conservation biological control strategy against *Tetranychus urticae* in clementine mandarin orchards. Crop Prot 30**:**1328-1333.
3. Stevens MM, Madge DG, James DG, Diffey S, Schiller LJ (2007) Ground cover management does not influence densities of key *Iridomyrmex* species (Hym.: Formicidae) in Australia citrus groves. J Appl Entomol 8: 532-536.
4. Paredes D, Cayuela L, Gurr GM, Campos M (2013) Effect of non-crop vegetation types on conservation biological control of pests in olive groves. PeerJ 1: e116.
5. Rodríguez E, González B, Campos M (2009) Effect of cereal cover crop on the main insect pest of Spanish olive orchards. J Pest Sci 82: 179-185.
6. Dong J, Wu X, Zhang Q, Jin X, Ding J, et al. (2005) Evaluation of lucerne cover crop for improving biological control of *Lyonetia clerkella* (Lepidoptera: Lyonetiidae) by means of augmenting its predators in peach orchards. Great Lakes Entomology 38: 186-200.
7. Wan NF, Ji XY, Jiang JX, Dan JG (2011) Effects of ground cover on the niches of main insect pests and their natural enemies in peach orchard. Chinese Journal of Ecology 30: 30-39.
8. Meagher RL, Meyer JR (1990a) Influence of ground cover and herbicide treatments on *Tetranychus urticae* populations in peach orchards. Exp Appl Entomol 9: 149-158.
9. McClure MS, Andreadis TG, Lacy HG (1982) Manipulating orchard ground cover to reduce the invasion by leafhopper vectors X-disease. J Econ Entomol 75: 64-68.
10. Meagher RL, Meyer RL (1990b) Effect of ground cover on certain abiotic and biotic interactions in peach orchard ecosystems. Crop Prot 9: 65-72.
11. Altieri MA, Schmidt LL (1985) Cover crop manipulation in Northem California orchards and vineyards: effects on arthropods communities. Biol Agric Hortic 3: 1-24.
12. Nyrop JP, Minns JC, Herring CP (1994) Influence of ground cover on dynamics of *Ambliseius fallacis* Garman (Acarina: Phytoseidae) in New York apple orchards. Agr Ecosyst Environ 50: 61-72.
13. Wyss E (1995) The effects of weed strips on aphids and aphidophagous predators in an apple orchard. Entomol Exp Appl 75: 43-49.
14. Stephens MJ, France CM, Wratten SD, Frampton C (1998) Enhancing biological control of leafrollers (Lepidoptera: Tortricidae) by sowing buckwheat (*Fagopyrum esculentum*) in an orchard. Biocontrol Sci Techn 8: 547-558.
15. Jenser G, Balázs K, Erdélyi CS, Haltrich A, Kádár F (1999) Changes in arthropods population composition in IPM apple orchards under continental climatic conditions in Hungary. Agr Ecosyst Environ 73: 141-154.
16. Irvin NA, Scarratt SL, Wratten SD, Frampton CM, Chapman RB, et al. (2006) The effects of floral understoreys on parasitism of leafroller (Lepidoptera: Tortricidae) on apples in New Zealand. Agr Forest Entomol 8: 25-34.
17. Marko V, Jenser G, Kondorosy E, Abraham L, Balazs K (2013) Flowers for better pest control? The effects of apple orchard ground cover management on green apple aphids (*Aphis* spp.) (Hemiptera: Aphididae), their predators and the canopy insect community. Biocontrol Sci Techn 23: 126-145.
18. Bone NJ, Thomson LJ, Ridland PM, Cole P, Hoffmann AA (2009) Cover crops in Victorian apple orchards: Effects on production, natural enemies and pests across a season. Crop Prot 28**:** 675-683.
19. Frechette B, Cormier D, Chouinard G, Vanoosthuyse G, Lucas E (2008) Apple aphid, *Aphis* spp. (Hemiptera: Aphididae), and predator populations in an apple orchard at the non-bearing stage: The impact of ground cover and cultivar. Eur J Entomol 105: 521-529.
20. Nicholls CI, Parrella MP, Altieri MA (2000) Reducing the abundance of leafrollers and thrips in a Northern California organic vineyard through maintenance of full season floral diversity with summer cover crop. Agric For Entomol 2: 107-113.
21. Costello MJ, Danne KM (2003) Spider and leafhopper (*Erythroneura* spp.) response to vineyard ground cover. Environ Entomol 32: 1085-1098.
22. English-Loeb G, Rhains M, Martinson T, Ugine T (2003) Influence of flowering cover crops on *Anagrus* parasitoids (Hymenoptera: Mymaridae) and *Erythroneura* leafhoppers (Homoptera: Cicadellidae) in New York vineyards. Agric For Entomol 5: 173-181.
23. Berndt LA, Wratten SD, Scaratt SC (2006) The influence of floral resource subsidies on parasitism rates of leafrollers (Lepidoptera: Tortricidae) in New Zealand vineyards. Biol Control 37: 50-55.
24. Williams L, Hain FP, Orr D (2011) Influence of four ground cover vegetation types used in North Carolina Fraser Fir Christmas tree plantations. J Entomol Sci 46: 216-222.
25. Fitzgerald JD, Solomon MG (2004) Can flowering plants enhance numbers of beneficial arthropods in UK apple and pear orchard? Biocontrol Sci Techn 14: 291-300.
26. Rieux R, Simon S, Defrance H (1999) Role of hedgerows and ground cover management on arthropod populations in pear orchard. Agr Ecosyst Environ 73: 129-140.
27. Beizhou S, Zhang J, Jinghui H, Hongying W, Yun K, et al. (2011) Temporal dynamics of the arthropod community in pear orchard intercropped with aromatic plants. Pest Manag Sci 67: 1107-1114.
