# Supplementary material for: Is Ground Cover Vegetation an Effective Biological Control Enhancement Strategy against Olive Pests?
Source: PLoS One. 2015 Feb 3;10(2):e0117265. doi: 10.1371/journal.pone.0117265 (PMC4315409; doi:10.1371/journal.pone.0117265)
Supplement: S2 Appendix — (DOCX) [file pone.0117265.s002.docx]

Appendix S2

| Table 1. Statistical descriptors of the number of observations per orchard and year before pairing. Q represents quartiles. | | | | | | | | | | | | |
| --- | --- | --- | --- | --- | --- | --- | --- | --- | --- | --- | --- | --- |
| Species | Response variable | Mean | Sd | Min | Max | Q0 | Q2.5 | Q25 | Q50 | Q75 | Q97.5 | Q100 |
| *P. oleae* |  |  |  |  |  |  |  |  |  |  |  |  |
|  | Funnel traps adults generation 1&2 | 14.14 | 3.21 | 6 | 41 | 1 | 3 | 11 | 14 | 17 | 18 | 41 |
|  | Larvae / inflorescence | 3.56 | 1.55 | 6 | 17 | 1 | 1 | 3 | 4 | 4 | 7 | 17 |
|  | Funnel traps adults generation 3 | 8.38 | 1.62 | 6 | 16 | 1 | 1 | 5 | 7 | 9 | 11 | 16 |
|  | Larvae / fruit | 4.44 | 2.21 | 6 | 29 | 1 | 1 | 3 | 4 | 6 | 9 | 29 |
| *B. oleae* |  |  |  |  |  |  |  |  |  |  |  |  |
|  | Funnel traps adults | 17.15 | 4.33 | 6 | 31 | 1 | 2 | 14 | 17 | 20 | 24 | 31 |
|  | Sticky traps adults | 16.48 | 4.00 | 6 | 34 | 1 | 5 | 14 | 17 | 19 | 24 | 34 |
|  | Damaged fruits | 14.37 | 4.96 | 6 | 40 | 1 | 2 | 12 | 15 | 18 | 22 | 40 |
| *E. olivina* | Nymphs / inflorescence | 5.6 | 5.4 | 6 | 38 | 1 | 1 | 2 | 4 | 7 | 22 | 38 |
| *S. oleae* | Living forms / shoot | 3.30 | 1.65 | 6 | 28 | 1 | 1 | 2 | 3 | 4 | 6 | 28 |

| Table 2. Correlation coefficient of peak abundance versus cumulative abundance for median and number of observations. | | | | |
| --- | --- | --- | --- | --- |
| Species | Response variable | Median | R | Number observations |
| *P. oleae* |  |  |  |  |
|  | Funnel traps adults generation 1&2 | 14 | 0.940 | 1256 |
|  | Larvae / inflorescence | 4 | 0.894 | 3129 |
|  | Funnel traps adults generation 3 | 7 | 0.937 | 1307 |
|  | Larvae / fruit | 4 | 0.916 | 2399 |
| *B. oleae* |  |  |  |  |
|  | Funnel traps adults | 17 | 0.773 | 1021 |
|  | Sticky traps adults | 17 | 0.859 | 1528 |
|  | Damaged fruits | 15 | 0.914 | 1184 |
| *E. olivina* | Nymphs / inflorescence | 4 | 0.960 | 533 |
| *S. oleae* | Living forms / shoot | 3 | 0.882 | 2752 |
